# Prevalence of auditory neuropathy spectrum disorder in an auditory health care service

**DOI:** 10.5935/1808-8694.20130077

**Published:** 2015-10-08

**Authors:** Rosimar Costa Penido, Myriam Lima Isaac

**Affiliations:** a PhD student, Otorhinolaryngology, School of Medicine of Ribeirão Preto, University of São Paulo, Department of Ophthalmology, Otorhinolaryngology and Head and Neck Surgery (Professor, Federal University of Maranhão, Center for Social Sciences, Health and Technology).; b PhD in Pediatric Medicine, School of Medicine of Ribeirão Preto (Professor, Department of Ophthalmology, Otorhinolaryngology and Head and Neck Surgery, School of Medicine of Ribeirão Preto, University of São Paulo).; School of Medicine of Ribeirão Preto - USP. Federal University of Maranhão - UFMA.

**Keywords:** evoked potentials, auditory, brain stem, hearing loss sensorineural, prevalence

## Abstract

Auditory neuropathy spectrum disorder (ANSD) is characterized by impairment of the auditory nerve associated with preservation of outer hair cell function.

**Objective:**

To establish the prevalence of ANSD in subjects with sensorineural hearing loss (SNHL).

**Method:**

This retrospective study was carried out between 2010 and 2012 and included the charts of 2,292 individuals with SNHL. Data from otolaryngological and audiological examinations based on pure-tone and speech audiometry, impedance tests, otoacoustic emissions (OAEs), and brainstem auditory evoked potentials (BAEPs) were collected. Inclusion criteria: presence of OAEs and/or cochlear microphonic (CM); absent or altered BAEPs, and normal MRI scans of the brain.

**Results:**

Twenty-seven (1.2%) of the 2,292 subjects with SNHL had ANSD (37% males; 63% females). Mild SNHL was seen in 29.6% of the individuals with ANSD; 55.5% had moderate SNHL; 7.4% had severe SNHL; and 7.5% had profound SNHL. In terms of age, 14.8% were aged between zero and 20 years, 44.1% were 41 to 60 years old, and 7.4% were above the age of 60.

**Conclusion:**

ANSD was seen in 1.2% of the individuals with SNHL included in this study.

## INTRODUCTION

The term auditory neuropathy has been used to describe diseases affecting children and adults characterized by normal outer hair cell function and anomalous or absent auditory nerve function; it has been described as a set of auditory disorders that combine otoacoustic emissions (OAEs) and/or cochlear microphonics (CM) with absent or desynchronous waves generated in brainstem auditory evoked potentials (BAEPs). The term auditory neuropathy was used for the first time in a study carried out in 1996 to categorize a group of subjects with hearing symptoms and normal cochlear function associated with cochlear nerve dysfunction^1^.

The term auditory neuropathy spectrum was produced through international consensus during the Guidelines Development Conference on the Identification and Management of Infants with Auditory Neuropathy, held in June of 2008^2^. The change in nomenclature was due to a study carried out in 2002 in which it was revealed that approximately half the children with auditory neuropathy had the same speech detection skills as children with sensorineural hearing loss, in addition to auditory brainstem responses, while the other half had unsatisfactory results in speech detection tests and no auditory brainstem responses. It was then realized that the term auditory neuropathy describes an array of disorders that may range from auditory dyssynchrony to auditory nerve neuropathy^3^.

Individuals with auditory neuropathy spectrum disorder (ANSD) may present mild to severe, unilateral or bilateral hearing loss associated with disproportional impairment of speech discrimination in relation to hearing loss^4^. BAEP testing also shows widely variable audiological results, from severely altered wave morphology to no wave formation, thus reflecting the multifaceted and heterogeneous nature of the pathophysiology of this auditory disorder^5^. Subjects with ANSD can often hear, but fail to discriminate words due to the loss of neural synchrony between the fibers of the vestibulocochlear nerve, thus severely impairing temporal auditory processing and speech discrimination^6^.

The published estimated prevalence rates of ANSD^7^ range from 0.23%^8^ to 15%^9^ in individuals with hearing loss. However, the estimated prevalence of ANSD in a study with patients at risk for hearing loss was 1.3%^10^, while a study with children at risk of hearing loss found a prevalence rate of ANSD of 0.94%^11^. Another study with children with hearing loss revealed prevalence rates ranging from 5.1% to 15%^12^. A study on sensorineural hearing loss found ANSD in 1.6%^13^ of the enrolled patients. A study with neonates screened for BAEPs found ANSD in 2.96%^14^ of the subjects. ANSD patients often require specific approaches to address their auditory, communication, and language impairments, which differ from the therapies proposed to patients with peripheral hearing loss^11^.

The site of injury in ANSD patients has not been clearly defined. Investigated sites include the inner hair cells, the synapses between inner hair cells and the auditory nerve, dendrites or neural axons, afferent and efferent activity of the auditory nerve, spiral ganglion neurons, and neurotransmitter biochemical anomalies^9^, ^15^, ^16^.

This study aimed to identify the prevalence of ANSD in a group of individuals with sensorineural hearing loss seen at an auditory health care center.

## METHOD

This cross-sectional historical cohort study was approved by the Research Ethics Committee of the institution (permit #190.328).

The study included subjects diagnosed with sensorineural hearing loss (SNHL) seen in an audiological care center from 2010 to 2012. The enrollment criteria included:

• SNHL;
• Ear endoscopic examination characterizing normal middle ear function;
• Transient otoacoustic emissions and/or cochlear microphonics in brainstem auditory evoked potential testing;
• Absent acoustic reflex;
• Absence of waves on BAEP testing or waves with severely altered morphology;
• Brain MRI scans ruling out retrocochlear diseases that could affect BAEP responses; MRI scans were made in other centers, therefore the reports were used to exclude patients only.

Exclusion criteria:

• Individuals with mixed or conductive hearing loss according to pure-tone and speech audiometry;
• Individuals not willing to cooperate during tests;
• Subjects with retrocochlear disease seen on MRI scans.

A total of 2,292 individuals diagnosed with sensorineural hearing loss with ages ranging from zero to 95 years were assessed. They underwent thorough assessment, comprised by ENT examination to find the status of the ear canal and the tympanic membrane through conventional otoscopy; basic audiological examination including impedance tests, pure-tone audiometry, and speech audiometry. The following devices were used: impedance testing device AT-235 (Interacoustics), audiometer AC-33 (Interacoustics).

OAEs were recorded with a Madsen Capela (Otometrics) device. Distortion product otoacoustic emissions were recorded at 700 Hz and 8000 Hz, and intensity of stimuli was kept fixed on L1 at 65 dBNPS and L2 at 55 dBNPS along with a ratio between frequencies of 1.22 (F2/F1 = 1.22). Response was interpreted as present in the frequencies in which the signal to noise ratio was 6 dB, reproducibility was equal to or greater than 70%, and stability was equal to or greater than 75%.

BAEPs and CM were assessed using device Chartr EP (Otometrics) with patients wearing in-ear earphones. Monaural clicks set initially at 100 dBNA were used at least twice in BAEP testing to confirm the presence of overlapping waves. Stimulus frequencies ranged from 250 Hz to 8000 Hz each lasting 100 microseconds; condensation and rarefaction clicks were presented 27.7 times per second with a 12 ms window. Absence of wave formation on BAEP testing with stimuli up to 100 dBNA and severe wave morphology alterations in BAEP consisting of low amplitude wave V at 100 dBNA were characterized. CM was recorded during BAEP testing using clicks with inverted polarity (condensation and rarefaction). When CM was present at 100 dBNA, intensities in decreasing steps of 20 dBNA were tested to the minimum level at which the potential could be verified. The absolute latencies for waves I, III, and V were measured and found to have varied significantly due to alterations in morphology, amplitude, and latency, but such measurements were not part of the goals of the study. BAEP and OAE tests were conducted without the use of sedatives. The classification proposed by Goodman^17^ was used to categorize SNHL.

## RESULTS

**Graph 1:**
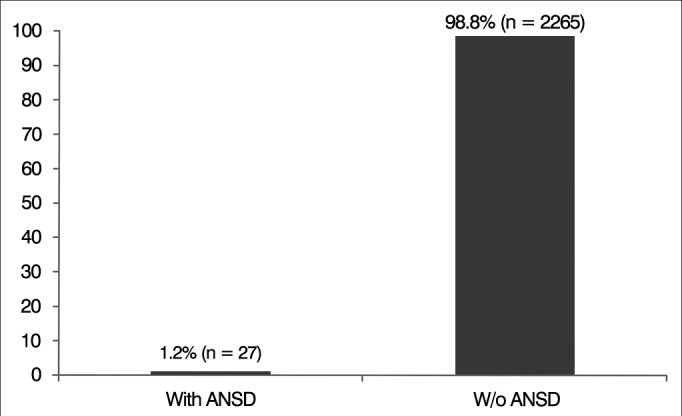
Percent distribution of subjects with auditory neuropathy spectrum disorder (ANSD).

Twenty-seven (1.2%) of the 2,292 subjects included in the study diagnosed with SNHL (Graph 1) met the criteria for ANSD. They had absent or severely anomalous waves on BAEP and OAE tests and/or normal CM, in addition to normal brain MRI scans. All 27 individuals had binaural ANSD.

**Graph 2:**
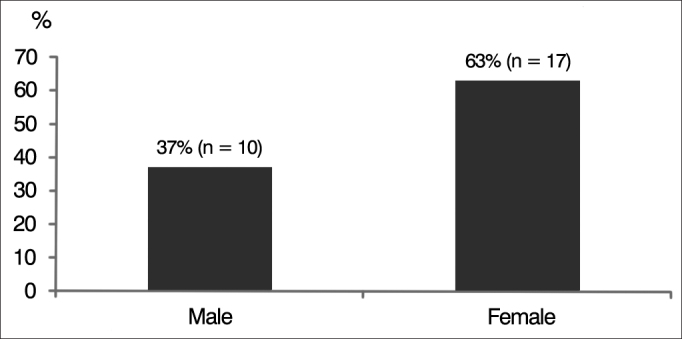
Gender percent distribution of subjects with auditory neuropathy spectrum disorder (ANSD).

Ten (37%) of the 27 subjects were males and 17 (63%) were females (Graph 2).

**Graph 3:**
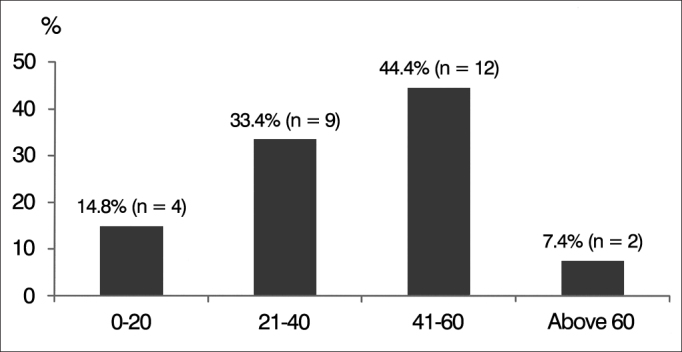
Age percent distribution of subjects with auditory neuropathy spectrum disorder (ANSD).

When age distribution was considered, four (14.8%) subjects were aged between zero and 20 years; nine (33.4%) were aged between 21 and 40; 12 (44.4%) were aged between 41 and 60; and two (7.4%) were above 60 (Graph 3).

**Graph 4:**
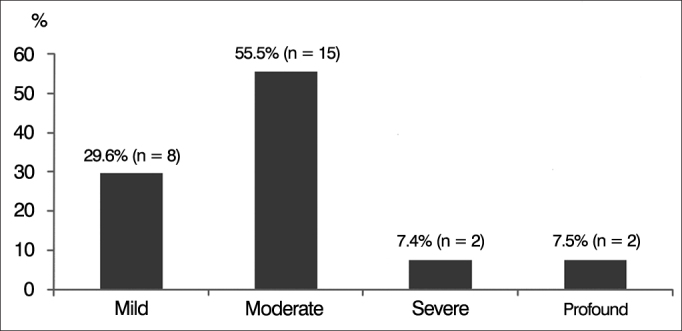
Percent distribution of subjects with auditory neuropathy spectrum disorder (ANSD) according to degree of hearing loss.

Pure-tone and speech audiometry indicated that eight (29.6%) subjects had mild SNHL; 15 (55.5%) had moderate SNHL; two (7.4%) had severe SNHL; and two (7.5%) had profound SNHL (Graph 4). Impedance tests revealed that none of the individuals had stapedial reflexes.

**Graph 5:**
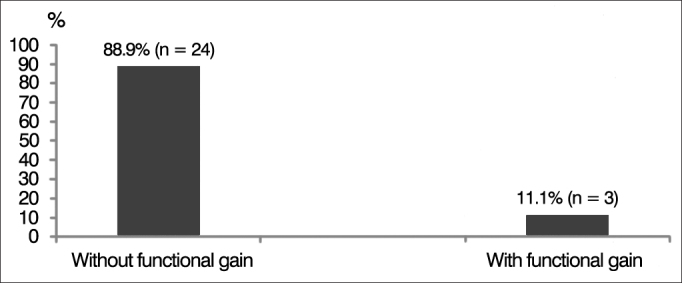
Percent distribution of subjects with auditory neuropathy spectrum disorder (ANSD) according to functional gain after fitting of hearing aids.

The 27 subjects diagnosed with ANSD were fitted with hearing aids. Functional gain was observed in three (11.1%) individuals, and 24 (88.9%) had no functional gain (Graph 5).

## DISCUSSION

Auditory neuropathy spectrum disorder is a condition found in patients of all ages with normal outer hair cell function and altered neural function^16^. The “mismatch” seen in the test results of ANSD patients reveal the neural synchrony alteration characteristically seen in this condition. The combination of objective and subjective audiological tests is required for accurate diagnosis and aid in the definition of patient prognosis. Recommended tests include pure-tone and speech audiometry; acoustic impedance; otoacoustic emissions; brainstem auditory evoked potentials; and cochlear microphonics^18^.

Cochlear microphonics can be assessed through BAEPs or electrocochleography in the diagnostic tests for ANSD. Studies on subjects with ANSD looked into the use of extratympanic electrocochleography (Et-Ecog) and BAEP testing in the differential diagnosis of ANSD, and concluded that Et-Ecog allows for more detailed analysis of cochlear function than BAEPs^19^.

ANSD is a relatively new clinical entity and there still is little evidence on its incidence, prevalence, natural history, and possible courses of therapy^20^.

The prevalence of ANSD in subjects with SNHL in our study was 1.2%. The literature contains reported prevalences ranging from 0.5% and 15% in subjects with SNHL^8^, ^9^, ^10^. Another study reported prevalences ranging from 1.8% to 14% in children with hearing loss^11^, ^12^. Other authors reported a prevalence rate of 5.1% of ANSD in children with SNHL^21^.

Studies carried out with adults showed lower prevalences of ANSD. Lofti & Mehrkian^13^ reported rates of 1.6%; Lee et al.^22^ found prevalences of 2.5%; and Duman et al.^23^ reported 4% prevalence rate.

Patients of all age ranges were found to have ANSD in this study, but adults accounted for most of the cases, with 23 of the 27 individuals diagnosed with ANSD.

ANSD progression and the damage to hair cells caused by the cochlear damage produced by the amplification provided by hearing aids in subjects with SNHL lead to the disappearance of OAEs and hamper the diagnosis of ANSD. Thus, cochlear microphonics become a relevant part of the tests used to diagnose patients with ANSD^24^.

In our study, all subjects diagnosed with ANSD met the criteria for preserved cochlear function associated with altered or absent auditory nerve function according to OAE and BAEP testing, respectively. Test results were consistent with the findings described in the literature in regards to gender distribution, age range, and degree of hearing loss^25^, ^26^, ^27^. Only three (11.6%) of the 27 individuals with ANSD had some functional gain after they were fitted with hearing aids, revealing the unsatisfactory outcome of this intervention.

Cochlear implants may improve the waves on BAEP testing and the speech of subjects with ANSD^28^, in addition to effectively improving the hearing of more than 90% of the affected individuals by compensating the neural synchrony alteration seen in ANSD and improving speech recognition^29^, ^30^.

ANSD is not an extremely rare auditory condition. The accurate diagnosis of this disease requires, in addition to audiological assessment, objective OAE, CM, and BAEP tests, as the presence of CM or OAEs associated with BAEP wave absence or dyssynchrony is a significant diagnostic criterion.

ANSD is a challenging condition, as many factors concerned with its pathogenesis and etiology are yet unclear. More studies are required to provide much needed clarification on ANSD.

## CONCLUSION

The prevalence of auditory neuropathy spectrum disorder in this study in subjects with sensorineural hearing loss was 1.2%, as also reported in the literature.
